# Vinyl ether maleic acid block copolymers: a versatile platform for tunable self-assembled lipid nanodiscs and membrane protein characterization

**DOI:** 10.1039/d5py00767d

**Published:** 2025-12-01

**Authors:** Muhammad Zeeshan Shah, Evelyn Okorafor, Nancy C. Rotich, Quinton Henoch, Ranjita Thapa Acharya, Richard C. Page, Gary A. Lorigan, Dominik Konkolewicz

**Affiliations:** a Department of Chemistry and Biochemistry, Miami University 651 E High St Oxford OH 45056 USA d.konkolewicz@miamioh.edu

## Abstract

Vinyl ether-maleic anhydride (VEMAn) copolymers were chain extended with *n*-butyl acrylate (*n*BA) and *tert*-butyl acrylate (*t*BA) blocks using reversible addition–fragmentation chain transfer (RAFT) copolymerization. Subsequently, the copolymers underwent hydrolysis to synthesize vinyl ether-maleic acid (VEMA) copolymers with different tail structures. The *n*BA block yielded VEMA extended with an acrylic acid (AA) block after hydrolysis. The *t*BA block gave VEMA extended with a mixture of *t*BA and AA blocks. This study investigates the effect of VEMA hydrophilicity/hydrophobicity and monomer structure in the second block on the formation and properties of self-assembled lipid nanodiscs. In particular, the size of the polymer–lipid discs and their interaction with a model membrane protein, KCNE1. The findings indicate that both AA and *t*BA/AA VEMA blocks yield lipid discs, however copolymers with *t*BA/AA blocks tend to form relatively larger lipid nanodiscs potentially due to steric differences in the copolymer tail. The change in hydrophobicity of VEMA block copolymers affects the resulting dimensions of lipid nanodiscs; similarly, the type of lipid also influences the size of lipid discs. Electron Paramagnetic Resonance (EPR) studies revealed that these block copolymers do not affect the structural dynamics of the KCNE1 protein, confirming their suitability for membrane protein studies in native-like environments. This study demonstrates the compatibility of VEMA-block copolymers with membrane protein systems by enabling control over the size of lipid discs. Furthermore, it provides insight into the self-assembly understanding of these lipid nanodiscs and their interactions with membrane proteins.

## Abstract 2

Impact of terminal block structure on the self-assembly of vinyl ether maleic acid copolymers with lipids is investigated. The results indicate that terminal block can fine tune the size of self-assembled nanodiscs between the polymers and lipids.

## Graphical abstract

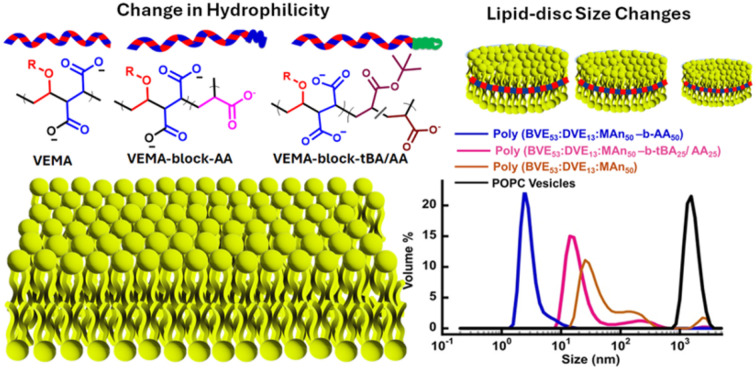

## Introduction

Polymers are broadly used in numerous applications, since they can be tailored towards emerging needs.^1,2^ Polymers are not limited to organic molecules for material properties but have also become essential towards biological applications.^3,4^ The properties and uses of polymers highly depend on their internal structure and the nature of the monomers used to synthesize them.^5,6^ Among these, copolymers are increasingly used in biological systems.^7,8^ Copolymers, as their name suggests, are usually made up of different monomers tailored to provide the properties of each monomer used in them.^9,10^ These copolymers can be synthesized through block/sequential,^11^ random,^12,13^ or alternating incorporation of monomers,^14,15^ depending on the nature of the monomers incorporated, the technique used for synthesis, and the desired properties for the application.^12–16^ The properties of copolymers can be further enhanced or tailored by extending the original chain with another polymer block.^17,18^ Block copolymers, due to their unique ability to combine the properties of distinct polymers, are widely used in drug delivery, material science, and nanotechnology.^19–21^

Synthesis of block copolymers can be carried out using living and living-like polymerization techniques, including reversible addition–fragmentation chain transfer (RAFT) polymerization.^1,22,23^ RAFT was first introduced in 1998, and is an excellent method for polymer synthesis due to its ability to synthesize low dispersity and controlled molecular weight polymers.^24^ Moreover, due to the technique's compatibility with a variety of monomers, it is widely being adopted for the synthesis of well defined functional polymers.^5,25^ The RAFT copolymerization process is generally initiated using conventional initiators that produce radicals which can add monomer.^24,26^ After radical production, the chain transfer agent (CTA) controls the homogeneity in size of chain lengths through the RAFT equilibrium.^24,27^

Amphiphilic copolymers have been used to develop systems that facilitate the study of membrane proteins in native environments.^28–30^ Membrane proteins are important for modern drug development, as these proteins are the target of more than 60% of FDA-approved drugs;^29,31,32^ however, studying membrane proteins has been challenging due to their complex structure and sensitivity to their environment.^33,34^

There are many membrane proteins which serve as stimulus controlled gated channels.^35^ The KCNE family of proteins is known to modulate the function of voltage-gated potassium channels.^36,37^ Malfunction of this protein has been reported to be associated with several diseases, including Long QT syndrome (LQTS), Jervell and Lange-Nielsen (JLN) syndrome, sudden cardiac death or arrhythmia, syncope, and congenital deafness.^35,37,38^ Incorrect modulation of these proteins can also provoke diseases like acute lymphoblastic leukemia.^39,40^ KCNE1 is a 15 kDa single-pass transmembrane protein with 170 amino acids.^40,41^ It modulates the function of KCNQ1 and forms a slow and delayed rectifier potassium channel.^39,42,43^ This potassium channel has a significantly important role in regulating the cardiac action potential.^37,39^ The importance of KCNE1 protein, and its well documented structure and functionality, makes it a good model system for studying membrane proteins.^41,44^

Traditional methods to study membrane proteins, including the KCNE family, include non-bilayer (micelles and amphipols)^45,46^ and bilayer systems (bicelles, membrane scaffold protein based lipid discs).^47–49^ Micelles and amphipols serve as poor analogues of native lipid membranes and can suffer from stability challenges.^50,51^ Bicelles have a bilayer structure surrounded by surfactant molecules, but their limitations include instability and the fact that only a few combinations of lipids can form them, hence they are not suitable for a wide range of membrane protein studies.^47,51^ Membrane scaffold protein (MSPs)-based nanodiscs are relatively stable bilayer structures.^52,53^ However, they have a major limitation in that the scaffolding protein can interfere with the analysis of the membrane protein of interest, and the relatively high cost of the MSPs can also present challenges to broader implementation.^52–54^

Polymer based lipid discs closely mimic the native membrane systems which are used to study membrane proteins.^33,55,56^ Styrene Maleic Acid (SMA) copolymers can form self-assembled lipid membrane particles called SMALPs.^57^ The aromatic ring in the styrene of SMALPs, however, can interfere with protein quantification.^58,59^ Aliphatic copolymers including α-olefin-maleic acid (αMAs) and diisobutylene maleic acid (DIBMA) copolymers can also form lipid nanodiscs while avoiding the limitations associated with other available systems.^59–65^ Vinyl ether-maleic acid (VEMA) copolymers, which generate lipid discs upon interaction with lipid molecules, have also been developed recently.^34^ These VEMA-based lipid particles (VEMALPs) have an advantage over traditionally used SMALPs due to the absence of an aromatic ring, as styrene absorbs UV light in the range of most proteins, which makes it difficult to analyze protein concentration using UV light.^34,59,62^ Furthermore, VEMALPs were found comparatively more stable than SMALPs at lower pH and high divalent ion concentrations.^34,59,64^ In comparison to diisobutylene maleic acid copolymer-based lipid discs (DIBMALPs), vinyl ether-maleic anhydride lipid particles (VEMALPs) offer the advantage of tunable solubility and nanodisc size by easily varying hydrophilic/hydrophobic balance by choice of available vinyl ethers, allowing polymers to be tuned to the protein and lipid of choice.^34,64,65^

The performance of polymer-based lipid discs is highly dependent on their internal hydrophilic–hydrophobic balance.^66,67^ Hydrophilic sites in SMA and VEMA copolymers are generated through the hydrolysis of maleic anhydride,^34,64,68^ resulting in the formation of hydrophilic maleic acid units in near perfect alternation with hydrophobic units of styrene or vinyl ether within the copolymer.^62^ This hydrophilic–hydrophobic balance can be tailored to develop the lipid discs of desired size and stability.^61,69^ The properties of lipid discs are also highly dependent on the interaction between lipid tails and the copolymer's hydrophobic units.^70,71^ Beyond the choice of hydrophobic unit length, by choice of the vinyl ethers, the hydrophobicity can be further tuned by the addition of a hydrophobic tail. Certain studies have shown that hydrophobic polystyrene tails in SMA can enhance performance.^69,72^ However, the impact of tails on lipid nanodisc properties in VEMA systems is unknown.

Here, VEMA copolymers would be extended by adding blocks of acrylic acid, and mixture of *tert*-butyl acrylate and acrylic acid. The incorporation of a new block at the tail of the VEMA copolymer will be achieved using RAFT chain extension polymerization. Previous studies indicate that the direct polymerization of acrylic acid presents difficulties due to the formation of Michael dimers,^73^ and potential exchange of the free carboxylic acid from AA and the anhydrides. Consequently, the *n*-butyl acrylate block will be used to achieve the incorporation of the acrylic acid block, with subsequent hydrolysis. In contrast, *tert*-butyl acrylate is more stable against base hydrolysis, compared to primary alcohol esters such as *n*BA,^74^ adding a larger number of hydrophobic ester units to the tail. Literature has shown that most acrylates are compatible with RAFT.^34^ Investigation of both mix of *tert*-butyl acrylate and acrylic acid, and acrylic acid allows the investigation of hydrophilic type effects on VEMA–lipid nanodisc properties.

## Experimental section

All materials were purchased commercially and were stored at appropriate temperatures as per vendor instructions unless otherwise specified. All reagents except vinyl ethers, solvents, and vinyl acrylates were obtained as solids. RAFT reagent 2-(propionic acid)yl dodecyl trithiocarbonate (PADTC) was synthesized in the lab using the procedure described in literature (Fig. S1).^75^

### Synthesis of vinyl ether-maleic anhydride (VEMAn) copolymer

VEMAn synthesis was performed using the method already available in literature.^34^ The monomers butyl vinyl ether (BVE), dodecyl vinyl ether (DVE), and maleic anhydride (MAn) with known ratios (described in Table 1) were mixed with the initiator azobis-(isobutyronitrile) (AIBN) and chain transfer agent (CTA) 2-(propionic acid)yl dodecyl trithiocarbonate (PADTC) in 25 mL flask. The 1,4-dioxane was added to this mixture (2 : 1 by mass). Monomer ratios used to synthesize the copolymers are described in Table 1. The solution was stirred to obtain homogeneous solution, and a small portion of sample was collected for one-dimensional proton Nuclear Magnetic Resonance (1D ^1^H NMR) to confirm the reactants before synthesis. The round bottom flask was then sealed with septa and nitrogen gas was purged through it for the next 30 minutes. After that, the nitrogen purge was stopped, and the solution was polymerized by heating it in an oil bath for 4 hours at 65 °C on a hot plate with a stirring rate of 220 rpm. After this process, a small portion of polymer was collected to perform post polymerization ^1^H NMR and size exclusion chromatography to confirm the polymerization. ^1^H NMR trace of copolymer is shown in Fig. S2.

**Table 1:** Monomer conversion and molecular weight for BVE/DVE/MAn copolymerization at 65 °C over a 4-hour duration

[BVE] : [DVE] : [MAn] : [PADTC] : [AIBN]	Monomer conversion		*M*_n_	*M*_nth_	*M*_w_/*M*_n_
	MAn	BVE + DVE			
53 : 13 : 50 : 1 : 0.3	∼93%	>95%	10 000	13 000	1.5
48 : 18 : 50 : 1 : 0.3	∼90%	∼90%	10 000	12 000	1.6
35 : 9 : 33 : 1 : 0.2	∼95%	>95%	7000	9000	1.3
212 : 52 : 200 : 1 : 1.2	∼56%	∼68%	28 000	33 000	1.7

### Precipitation of VEMAn and VEMAn-*block*-butyl acrylate (VEMAn-*block*-BA) copolymers

A 100 mL beaker with 80 mL of ice cold hexanes was placed in an ice bucket. A minimal amount of tetrahydrofuran (THF) was added to the copolymer and then polymer was transferred to hexanes beaker in dropwise manners. After visually confirming the precipitate, the excess solvent was poured out and the beaker was shifted to vacuum desiccator for drying purpose. The top of the beaker was first sealed with parafilm, and multiple holes were created with the help of a regular needle to facilitate the drying process. The polymer was vacuum dried for 24 hours. After 24 hours if the polymer was still not completely dry, the polymer was kept for another 24 hours. The same precipitation method was repeated after adding the second block.

### Synthesis of VEMAn-*block*-BA copolymer

**Fig. 1:**
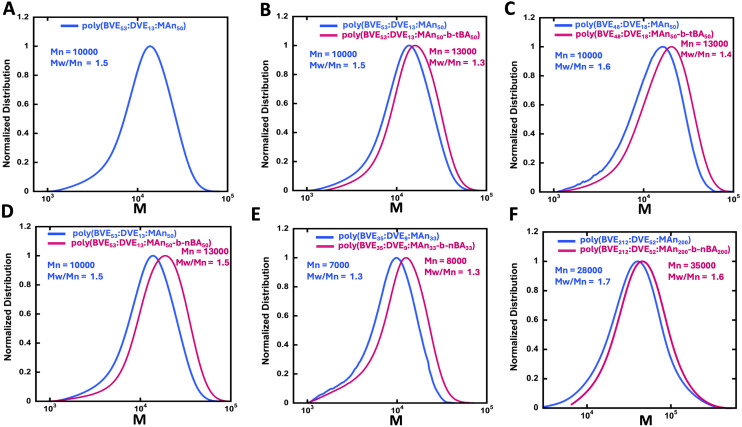
GPC traces of copolymers and block extensions (A) poly(BVE_53_ : DVE_13_ : MAn_50_) (B) poly(BVE_53_ : DVE_13_ : MAn_50_-*b-t*BA_50_) (C) poly(BVE_48_ : DVE_18_ : MAn_50_-*b-t*BA_50_) (D) poly(BVE_53_ : DVE_13_ : MAn_50_-*b-n*BA_50_) (E) poly(BVE_35_ : DVE_9_ : MAn_33_-*b-n*BA_33_) (F) poly(BVE_212_ : DVE_52_ : MAn_200_-*b-n*BA_200_).

After drying the first block copolymer VEMA in vacuum, the solid copolymer was added with 50 molar excess *t*-butyl acrylate/*n*-butyl acrylate and 30% AIBN with respect to CTA. The solution was purged for 30 min with nitrogen gas in a 25 mL round bottom flask in identical volume of 1,4-dioxane used to synthesize the first block. After that, the polymerization reaction was run overnight at 65 °C using 220 rpm. After polymerization of VEMA-*block-t*BA/VEMA-*block-n*BA copolymers, size exclusion chromatography was performed, and comparison was studied (Fig. 1).

### Hydrolysis of copolymers

The copolymer was mixed with minimal THF and transferred to 100 mL round-bottom flask, stirred at 220 rpm at room temperature. 25 mL of water was then added per every gram of copolymer. 4 molar excess of sodium hydroxide was then added to the copolymer dropwise. The flask was then set in an oil bath at 50 °C and 220 rpm for 24 hours.

### Preparation of KCNE1

The KCNE1 mutant S74C was produced by overexpressing it in BL21 DE3 *Escherichia coli* cells (Stratagene). These cells were cultured in TB minimal medium supplemented with 50 μg mL^−1^ chloramphenicol and 75 μg mL^−1^ ampicillin. The cells were induced with 1 mM isopropyl-1-thio-d-galactopyranoside after growing the cell culture until an OD_600_ of 0.4–0.8 was reached. The protein was purified using the method published in literature^44^ with the exception that 0.5% dodecylphosphocholine (DPC) was used instead of 1-myristoyl-2-hydroxy-*sn-glycero*-3-phospho-(1′-*rac*-glycerol) (LMPG). The protein sample was concentrated using an Ultracel regenerated cellulose membrane concentrator of 3 kDa molecular weight cutoff (Millipore Sigma). Sodium dodecyl sulfate-polyacrylamide gel electrophoresis (SDS-PAGE) was then performed to verify the purity of protein.

### MTSL spin labeling

MTSL was purchased from Toronto Research Chemicals. 250 mM of MTSL in DMSO was added to the protein in pH 7.0 phosphate buffer (50 mM) and 0.05% DPC (MTSL : protein = 10 : 1). The solution was kept at room temperature for 30 minutes, followed by incubation at 37 °C while shaking for 3 hours, and finally, it was shaken at room temperature overnight. The labeled protein was then buffer exchanged into 25 mM phosphate pH 7.0 and 0.05% DPC by three rounds of centrifugation at 6000*g* in Amicon spin filter tubes (10 000 Da molecular weight cutoff). 7 mL of the resulting mixture was incubated overnight at 4 °C with 3 mL of Ni(ii)-NTA superflow resin (pre-equilibrated). The resin was then washed with a phosphate buffer (300 mL) at neutral pH and 0.05% DPC. The protein was then eluted with 8 mL of 50 mM phosphate buffer at neutral pH containing 250 mM imidazole, and 0.5% DPC.

### Vesicle reconstitution

A pear-shaped flask was used to create the lipid thin film by evaporation following the dissolving of solid lipids (1-palmitoyl-2-oleoyl-*sn-glycero*-3-phosphocholine (POPC) : 1-palmitoyl-2-oleoyl-*sn-glycero*-3-phospho-(1′-*rac*-glycerol) (sodium salt) (POPG) = 3 : 1) in chloroform. The mixture was then desiccated for 12 hours. A 50 mM of phosphate buffer at neutral pH was then added to the mixture to make 100 mM lipid solution. The fixed mixing ratio (1 : 400 for protein : lipid) was used to generate protein containing vesicles. The mixture underwent five freeze thaw cycles to generate proteoliposomes with protein concentration of approximately 100 μM. Dialysis techniques were used to remove any extra detergent for a period of 72 hours using 12–14 kDa tubes containing 100 mM imidazole with 2 mM EDTA at neutral pH which was changed every 12 hours. After dialysis, the mixture was centrifuged at 300 000*g* for half an hour. The resulting proteoliposome was reconstituted with a 50 mM phosphate buffer at neutral pH.

### Preparation of POPC, POPG and DMPC vesicles

A protocol already published was repeated to synthesize POPC, POPG, and (dimyristoylphosphatidylcholine) DMPC vesicles. Corresponding powdered lipid was dissolved using chloroform and a thin layer was created using evaporation technique in heart shaped flask. The sample was then kept in a vacuum desiccator for complete drying overnight. 20 mM *N*-(2-hydroxyethyl)piperazine-*N*′-ethanesulfonic acid (HEPES) buffer with 100 mM sodium chloride at neutral pH was used to dissolve the lipid thin layer. The solution underwent a freeze thaw process that was repeated five times. The solution was then mixed by vortexing for 30 seconds in between every freeze thaw cycle. Vesicles were then placed in cold storage at −20 °C.

### Copolymer and vesicle mixing protocol

Following a previous protocol in literature,^34^ 20 mM of HEPES buffer with 100 mM of NaOH was used at neutral pH to dissolve the copolymer to get approximately 5% (m/v) concentration. For homogeneous mixing of copolymer, samples were sonicated for half an hour. Copolymer solution was added dropwise to the corresponding lipid vesicles to maintain polymer to lipid ratio of 2 : 1. Samples were then subjected to three freeze thaw and sonication cycles. This mixing protocol was different than previously made VEMA^34^ where the number of freeze thaw cycles was five and sample were kept overnight for mixing and equilibration. Afterwards, samples were set up on the shaker and kept in −4 °C overnight. The mixture was then centrifuged under 25 000 rpm for 30 min before DLS measurements.

## Results and discussion

**Scheme 1:**
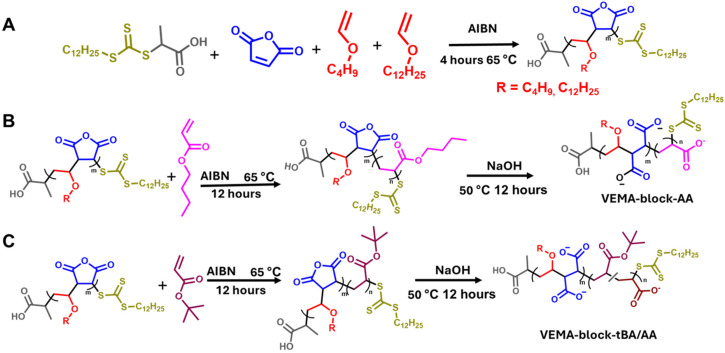
(A) Synthesis of VEMAn copolymers using RAFT copolymerization of vinyl ether and maleic anhydride monomers (B) addition of *n*-butyl acrylate block in VEMAn and hydrolysis to synthesize VEMA-*block*-AA polymers (C) addition of *tert*-butyl acrylate block in VEMAn and hydrolysis to synthesize the mixture of VEMA-*block-t*BA and VEMA-*block*-AA polymers.

RAFT polymerization was used to synthesize all VEMA polymers and block copolymers. Since RAFT homopolymerization of vinyl ethers is extremely challenging due to very low reactivity (∼zero),^34^ chain extension with hydrophobic acrylic monomers was performed. All reactions were performed at 65 °C for four hours. In all polymer synthesis reactions, the internal ratio of combined vinyl ethers (butyl vinyl ether (BVE) and dodecyl vinyl ether (DVE)) to maleic anhydride (MAn) was maintained at 66 : 50 to achieve a monomer internal ratio within the copolymer close to an alternating structure, while allowing near full conversion of all monomers.^34^ PADTC was employed as the CTA since it gives VEMAn polymers with relatively narrow molecular weight distributions.^34^ Polymers are denoted as poly(BVE_*x*_ : DVE_*y*_ : MAn_*z*_) for the initial VEMAn block and after chain extension with either *tert*-butyl acrylate (*t*BA) or *n*-butyl acrylate (*n*BA) polymers are denoted as poly(BVE_*x*_ : DVE_*y*_ : MAn_*z*_-*b-t*BA_*w*_) and poly(BVE_*x*_ : DVE_*y*_ : MAn_*z*_-*b-n*BA_*w*_) respectively, where *x*, *y*, *z*, and *w* represent the number of monomer units (Scheme 1).

Changes in the ratio of BVE to DVE monomers controlled the hydrophobicity of the copolymer, since DVE is substantially more hydrophobic than BVE. In addition, altering the ratio of total monomers to CTA changed the primary chain length. The theoretical and experimentally determined molecular weights of the VEMAn blocks are given in Table 1. In general, good agreement between the experimentally determined number average molecular weight (*M*_n_) from gel permeation chromatography (GPC) and the theoretical value was seen, with dispersity values (*M*_w_/*M*_n_) in the range of 1.3–1.6. As expected, the ratio of CTA to total monomers inversely affected the average molecular weight results from GPC. In most cases monomer conversion of each monomer was over 90%, suggesting high reaction efficiency, with the majority of the monomer being transformed into the polymer under these conditions. The higher dispersity in the order of *M*_w_/*M*_n_ ∼ 1.5 is potentially indicative of less efficient RAFT exchange,^76^ plausibly due to the significant difference in monomer reactivity of the VE and MAn monomers, since prior work indicated that similar polymers showed excellent chain extension efficiency and livingness.^34^

After the synthesis of poly(BVE : DVE : MAn) copolymers with distinct molecular weights, they were chain extended using either *t*BA or *n*-BA (Scheme 1). Two types of acrylate extensions, utilizing *t*BA and *n*BA, were chosen. *n*BA undergoes hydrolysis more readily than *t*BA due to reduced steric hindrance during base-catalyzed hydrolysis.^77^ This characteristic is helpful for obtaining the desired copolymer, VEMAn-acrylic acid, which will be subsequently denoted as (BVE_*x*_ : DVE_*y*_ : MAn_*z*_-*b*-AA_*w*_). All reactions involved the addition of 50 molar excess of tBA or nBA to CTA and they were conducted overnight at 65 °C. In all cases, the GPC traces show a shift in the molecular weight distribution towards higher molecular weight, indicating successful chain extension (Fig. 1).

In general, the relatively more compact *tert*-butyl groups give a relatively similar apparent chain length (Fig. 1B) to *n*BA (Fig. 1D). Increased hydrophobicity, achieved by incorporating a relatively higher ratio of DVE into the polymer (Fig. 1C) as compared to similar *t*-butyl acrylate extension (Fig. 1B), resulted in similar lower molecular weight increase upon chain extension.

After the synthesis of the five VEMAn block copolymers, they were subjected to hydrolysis to incorporate hydrophilicity into the copolymer through the opening of the anhydride ring (Scheme 1). Hydrolysis creates nearly perfectly alternating hydrophobic and hydrophilic sites in the VEMA segments, due to the strong tendency for alternation of vinyl ethers and maleic anhydride monomers along the backbone.^78–81^ To hydrolyze the anhydride ring, the polymers were reacted with NaOH in an aqueous solution at 50 °C for 12 hours. All synthesized block copolymers dissolved completely in aqueous solution (pH = 13) after overnight reaction, and they were subsequently dialyzed. Dialysis ultimately helped to neutralize the polymer (pH = 7) and remove impurities. The resulting hydrolyzed block copolymers were confirmed using IR spectroscopy (Fig. S4). It was also confirmed through NMR that *t*-BA underwent partial hydrolysis (∼50%) while *n*BA underwent essentially complete hydrolysis (Fig. S3).

**Fig. 2:**
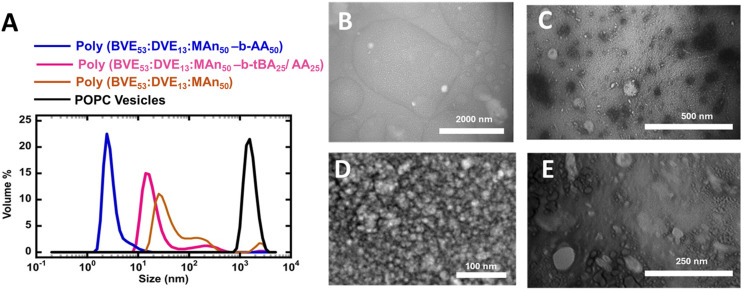
(A) DLS size distribution comparison of POPC vesicles and polymer lipid discs based on poly(BVE_53_ : DVE_13_ : MA_50_-*b-t*BA_25_/AA_25_), poly(BVE_53_ : DVE_13_ : MA_50_-*b*-AA_50_) and poly(BVE_53_ : DVE_13_ : MA_50_) in POPC lipids. TEM images of (B) POPC vesicles (C) poly(BVE_53_ : DVE_13_ : MA_50_) based lipid discs (D) poly(BVE_53_ : DVE_13_ : MA_50_-*b*-AA_50_) based lipid discs (E) poly(BVE_53_ : DVE_13_ : MA_50_-*b-t*BA_25_/AA_25_) based lipid discs.

Subsequently, these copolymers were lyophilized and then mixed with lipids (Fig. 2) to form various types of self assembled lipid discs.

The self-assembly of polymers with lipids to give lipid discs was investigated using dynamic light scattering. Fig. 2A illustrates the size distribution of VEMA poly(BVE_53_ : DVE_13_ : MA_50_) copolymer and its block extensions with *t*BA poly(BVE_53_ : DVE_13_ : MA_50_-*b-t*BA_25_/AA_25_), and AA poly(BVE_53_ : DVE_13_ : MA_50_-*b*-AA_50_) chain based lipid discs in POPC lipids, compared to the size distribution of POPC vesicles.

The poly(BVE_53_ : DVE_13_ : MA_50_) copolymer interacts with lipids due to its amphiphilic nature, resulting in self-assembly into disc-shaped structures. POPC vesicles had an approximate size of 1500 nm, while the polymers clearly interact with the lipids to give nanodiscs in the range of 3–30 nm. Change in hydrophobic–hydrophilic balance and electrosteric stabilization^82^ through the incorporation of either *t*BA/AA or AA blocks at the end of VEMA copolymer resulted in a narrow size distribution. Furthermore, the incorporation of a hydrophobic blocks of *t*BA or hydrophilic block of AA at the tail of the VEMA copolymer leads to a reduction in the size of the resulting lipid discs to ∼10 nm for *t*BA/AA and ∼3 nm for AA, compared to VEMA-based lipid discs without block attachments size ∼30 nm. This study clarifies that the nature of tails at the end of the copolymer plays an important role in determining the size of the resulting nanodiscs. These results suggest that the *t*BA/AA or AA copolymer tails interact favorably with POPC lipids, possibly even enhancing disc stability, leading to the narrow size distribution observed in the resulting lipid discs, and smaller particle sizes. Due to lower steric constraints, the hydrophylic AA tail stabilizes the lipid disc, possibly by electrosteric stabilization, leading to smaller particle size.^82^ In the *t*BA/AA polymer, the tertiary butyl group that is in the ester is expected to be too bulky to effectively intercalate with lipids, therefore we also expect the *t*BA/AA block to act as an electrosteric stabilizer. However, due to the higher anionic charge density, the AA block is expected to be a more effective electrosteric stabilizer than *t*BA/AA, plausibly explaining the more uniform and smaller particle size in the AA system compared to the *t*BA/AA system. To further explore this phenomenon, transmission electron (TEM) microscopy was employed to confirm the structure and size of the discs, (Fig. 2B–E, Fig. S10–11). Transmission electron microscopy data confirms that in all three types of copolymers and POPC lipid vesicles interactions, self-assembled nanodiscs were formed in the ∼10 nm diameter range.

**Fig. 3:**
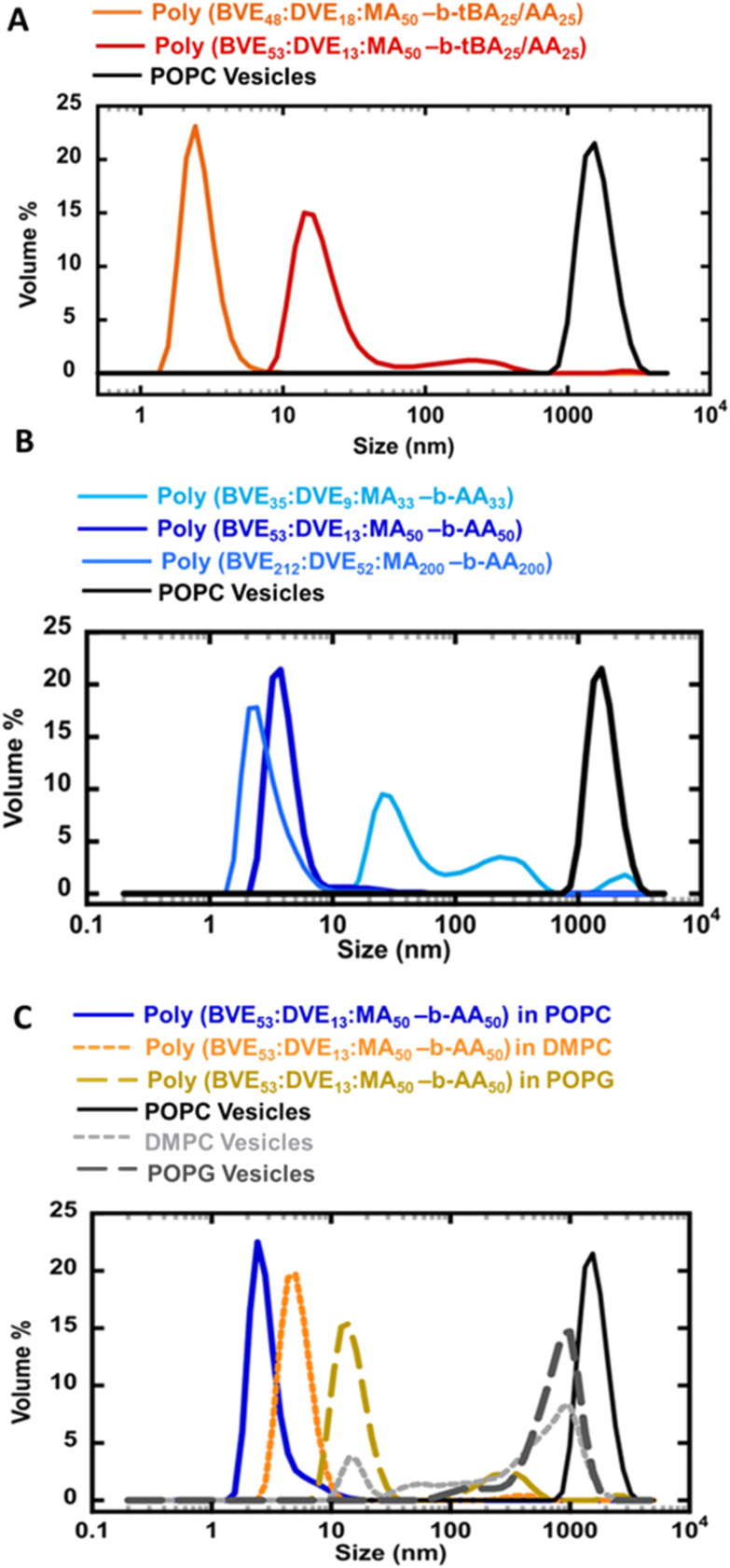
DLS size distribution (A) comparing effect of hydrophobicity in VEMA block copolymers (B) comparing effect of chain length in VEMA block copolymers (C) DLS size distribution comparing effect of lipids type in VEMA block copolymers.

The impact of hydrophobicity of the VEMA block was also investigated. In addition to the previously studied VEMA polymer, poly(BVE_53_ : DVE_13_ : MA_50_-*b-t*BA_25_/AA_25_), was compared against a copolymer with similar molecular weight, but more hydrophobic units of DVE relative to BVE, poly(BVE_48_ : DVE_18_ : MA_50_-*b-t*BA_25_/AA_25_). GPC confirmed that the first blocks of both the VEMA copolymers, poly(BVE_53_ : DVE_13_ : MAn_50_) and poly(BVE_48_ : DVE_18_ : MA_50_), had similar *M*_n_ values of ∼10 000, and as seen in Fig. 1 chain extension in both systems was successful. These VEMA-*block-t*BA/AA copolymers were then self-assembled with POPC lipids for a comparative analysis of the size distribution of self-assembled lipid discs (Fig. 3A). Fig. 3A demonstrated that the increasing hydrophobicity in first block of VEMA copolymer by incorporating higher DVE ratio caused a shift in particle size from ∼30 nm for poly(BVE_53_ : DVE_13_ : MA_50_-*b-t*BA_25_/AA_25_) to ∼3 nm for poly(BVE_48_ : DVE_18_ : MA_50_-*b-t*BA_25_/AA_25_). This is consistent with the superior anchoring of the more hydrophobic DVE units; however, as seen in earlier studies, using even higher ratios of DVE leads to polymers that are challenging to suspend in water, reducing the efficiency of VEMALPs formation.^83^ Overall, the results from Fig. 2 and 3A indicate that modifications to either the hydrophobicity of the VEMA block, or the AA containing second block significantly influence lipid disc size, with discs in the order of 3 nm possible from various approaches. The use of a more hydrophobic VEMA block, containing more DVE, with the less efficient *t*BA/AA hydrophobic tail suggests an alternative strategy for controlling lipid disc nature, providing multiple pathways to control the size of the self assembled disc.

Beyond the effect of hydrophobicity, the effect of the average molecular weight and chain length of the first block in VEMA-*block*-AA lipid discs were studied after creating polymers with variable sizes of the first VEMA block of 7000, 10 000, and 28 000, followed by chain extension with units of nBA. Same molar excess of *n*-butyl acrylates was ensured for the block copolymer reaction as the initial maleic anhydride units relative to CTA in the first block. These acrylates were subsequently converted to AA during hydrolysis. As seen in Fig. 3B, the shortest VEMA block copolymer, poly(BVE_35_ : DVE_9_ : MA_33_-*b*-AA_33_), after self assembly with POPC resulted in lipid nanodiscs exhibiting the largest size and broad size variance compared to those with higher molecular weight VEMA blocks; however, there was only a minimal difference in the sizes and breadth of the distribution between the two higher molecular weight block copolymers, poly(BVE_53_ : DVE_13_ : MA_50_-*b*-AA_50_) and poly(BVE_212_ : DVE_52_ : MA_200_-*b*-AA_200_). In general, VEMALPs derived from higher molecular weight VEMA block copolymers were smaller and more narrowly distributed. These results indicate that a change in the molecular weight of the initial VEMA block within VEMA-*block*-AA copolymers can serve as a tool to engineer lipid discs of varying sizes, thereby providing a potential way for tailoring lipid disc stability. The lower molecular weight VEMA block has fewer VE anchoring units per chain, perhaps resulting in weaker interactions with hydrophobic lipid tails and less efficient binding to the lipids. The increase in size and molecular weight of the initial block in VEMA-*block*-AA copolymers are directly correlated with an increase in the number of anchoring sites, which can facilitate initial intercalation of the VEMA segments into the lipids. This has been seen in SMA, where very low molecular weight polymers are less efficient at forming SMALPs.^84^ However, very large VEMA blocks will have more steric hinderance and conformational restrictions, as well as less efficient diffusion and possibly even lower solubility in the aqueous phase, thereby decreasing the efficiency of intercalation into the lipids.

The ability of poly(BVE_53_ : DVE_13_ : MA_50_-*b*-AA_50_) copolymers to form lipid discs was studied for a range of lipids (Fig. 3C). Specifically, the influence of overall chain length of the lipid hydrophobic tails and saturation was studied by comparing longer-chain unsaturated POPC lipids with shorter-chain saturated DMPC lipids. Furthermore, the effect of headgroup charge was studied through a comparison of zwitterionic POPC and DMPC lipids with negatively charged POPG lipids. Interestingly, self-assembling zwitterionic lipids POPC and DMPC with VEMA-*block*-AA copolymers yielded smaller size lipid nanodiscs (<10 nm) compared to lipid structures formed in POPG lipids (>30 nm) (Fig. 3C). The larger nanodisc size observed with POPG compared to POPC and DMPC can be attributed to the headgroup charge of the lipids. The anionic headgroup of negatively charged POPG may induce electrostatic repulsion, potentially hindering the interaction of hydrolyzed VEMA block copolymers, therefore resulting in larger nanodisc sizes compared to those formed with zwitterionic lipids. The potentially smallest size of nanodiscs formed between poly(BVE_53_ : DVE_13_ : MA_50_-*b*-AA_50_) and POPC lipids, compared to DMPC lipids, can be attributed to the presence of a *cis* unsaturated bond in the POPC lipid's tail. The unsaturated groups within POPC could cause looser packing of the lipids,^85,86^ thereby facilitating copolymer access for lipid disc formation. Although there was variation in disc size, based on the lipid used, the polymers were broadly applicable across a range of lipids, indicating that it is feasible to make nanodiscs using VEMA block copolymers, for a range of lipids.

**Fig. 4:**
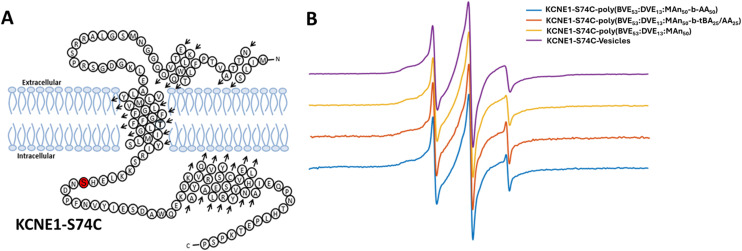
(A) Structures of KCNE1 protein with the location of attached spin labels.^83^ (B) CW-EPR spectra of the KCNE1 S74C spin-labeled system using VEMA copolymer block copolymers with either AA or mixture of *t*BA/AA, as well as VEMA copolymer and vesicles.

The effect of synthesized VEMA block copolymers on membrane protein structure was studied using Continuous Wave Electron Paramagnetic Resonance (CW-EPR). KCNE1 protein-S74C was spin-labeled and used as a model protein in this study (Fig. 4A). CW-EPR line spectra of KCNE1 have been well studied in vesicles and styrene maleic acid-based lipid copolymers based lipid discs.^87,88^ The lipid nanodiscs incorporating the KCNE1 protein, formed using the three copolymers (poly(BVE_53_ : DVE_13_ : MA_50_), poly(BVE_53_ : DVE_13_ : MA_50_-*b-t*BA_25_/AA_25_), and poly(BVE_53_ : DVE_13_ : MA_50_-*b*-AA_50_)) were subjected to CW-EPR analysis. All 3 systems yielded very similar line shape spectra to the vesicle lineshape (Fig. 4B). These results show that all studied copolymer systems, including VEMA lipid discs, VEMA-*block*-AA-based lipid discs and VEMA-*block-t*BA/AA-based lipid discs, had no significant effect on the structural dynamics of the KCNE1 protein, facilitating its use in applications for a range of model proteins.

## Conclusion

In this study, vinyl ether-maleic acid (VEMA) copolymers were successfully synthesized *via* reversible addition–fragmentation chain transfer (RAFT) polymerization and subsequently modified through the attachment of hydrophilic acrylic acid (AA) and hydrophobic *tert*-butyl acrylate (*t*BA) blocks. All synthesized copolymers formed self-assembled lipid nano-discs upon interaction with lipids. The block type at the copolymer termini affected the size of the assemblies. Specifically, AA blocks resulted in smaller lipid discs than *t*BA/AA blocks. Alterations in hydrophobicity, chain length, or the type of lipids influenced the size of self-assembled lipid nanodiscs. These amphiphilic block copolymers were compatible with the membrane protein, KCNE1, preserving the structural integrity of this membrane protein. This investigation yields insights into the creation of lipid nanodiscs with tailored size and helps in understanding the factors influencing the self-assembly of these block copolymers.

## Author contributions

M. Z. S. was involved in designing experiments, data acquisition, data analysis, manuscript writing, and editing. E. A. O. and N. C. R. were involved in experimental design, data acquisition, and editing. Q. H. was involved in data acquisition, analysis, and editing. R. A. was involved in data acquisition and data analysis. R. C. P. was involved in conceptualization, experimental design, and analysis. G. A. L. was involved in conceptualization, experimental design, and formal analysis. D. K. was involved in conceptualization, experimental design, formal analysis, writing, and editing.

## Conflicts of interest

There are no conflicts to declare.

## Supplementary Material

PY-017-D5PY00767D-s001

## Data Availability

Data for this article are available from the Miami University Scholarly Commons at: https://hdl.handle.net/2374.MIA/7041. Supplementary information is available. Supplementary information includes additional experimental protocols, polymer NMR data, polymer IR data, supplemental DLS data and correlation plots, block copolymer DOSY NMR data, lipid structures, supplemental TEM data. See DOI: https://doi.org/10.1039/d5py00767d.
