# Supplementary material for: Vinyl ether maleic acid block copolymers: a versatile platform for tunable self-assembled lipid nanodiscs and membrane protein characterization
Source: Polym Chem. 2025 Dec 1;17(2):194–206. doi: 10.1039/d5py00767d (PMC12679354; doi:10.1039/d5py00767d)
Supplement: PY-017-D5PY00767D-s001 [file PY-017-D5PY00767D-s001.pdf]

Supplementary Information (SI) for Polymer Chemistry.
This journal is © The Royal Society of Chemistry 2025

Supplementary material: Vinyl Ether Maleic Acid Block Copolymers: A Versatile Platform for Tunable Self-Assembled Lipid Nanodiscs and Membrane Protein Characterization

Muhammad Zeeshan Shah,[a] Evelyn Okorafor,[a] Nancy C. Rotich,[a] Quinton Henoch,[a] Ranjita Thapa Acharya,[a] Richard C. Page,[a] Gary A. Lorigan,[a] Dominik Konkolewicz * [a]

* Corresponding author

DK: d.konkolewicz@miamioh.edu

[a] Department of Chemistry and Biochemistry, Miami University, 651 E High St, Oxford, OH, 45056, USA

Supplemental Experimental Details

PADTC Synthesis:

A previously published method was used for PADTC synthesis.[1,2] In a 2.0L flask 56.25 g dodecane-1-thiol (DDT), 3.5572 g tetramethylammonium bromide (TMAB) and 900mL Acetone was added and stirring was started. At room temperature the solution was stirred until it was dissolved completely. After that, 11.25 g sodium hydroxide was added dropwise in the solution. The solution was left stirring for 15-20 minutes. The solution was put on ice until it became cold before adding 17.1g dropwise carbon disulfide which turned the color of solution to bright yellow. The reaction mixture was stirred for 30 minutes and then 43g 2-bromopropanoic acid was added dropwise which turned the solution color from cloudy orange to homogeneous orange. The reaction was stirring at 220 rpm overnight at room temperature. 1M HCl was added to the solution until yellow precipitate appeared. These precipitates were vacuum filtered and then added to the hexanes and heated in an oil bath until they completely dissolved. The solution was cooled to room temperature and then placed in the freezer for recrystallization. NMR was performed to confirm successful synthesis of the 2-(((dodecylthio)carbonothioyl)thio)propanoic acid (PADTC).

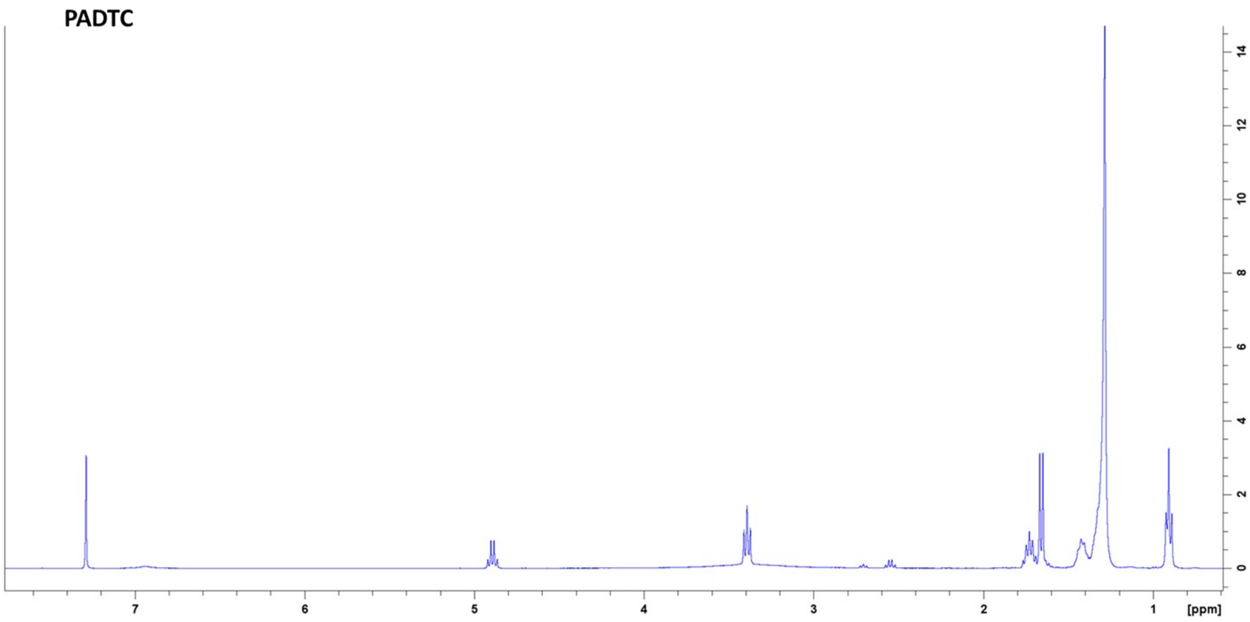

Figure S1. Nuclear Magnetic Resonance (1D ^{1}H NMR) spectra of 2-(((dodecylthio)carbonothioyl)thio)propanoic acid (PADTC)

SEC Conditions:

An Agilent GPC system 1260 or TOSOH EcoSEC Elite HLC-8420GPC equipped with a refractive index detector were used to determine the weight averages and dispersities of the polymers. A calibration curve was first generated using commercially available polymethyl methacrylate standards over the molecular weight range 617,000 to 1,010. Samples were prepared by dissolving 40-50 mg of copolymer in 1 mL of THF using toluene as a flow rate marker, followed by filtration through nanofilters and transferring to a 2 mL GPC vial. The samples were then run through the column using THF as the mobile phase, and the polymer peaks were subsequently integrated.

Supplemental Data

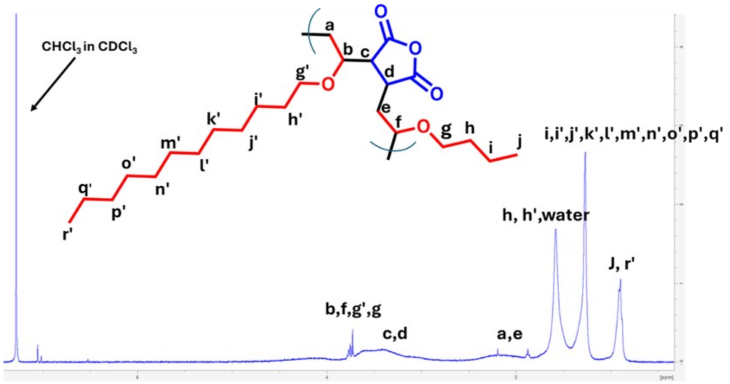

Figure S2. Proton Nuclear Magnetic Resonance (proton-NMR) of VEMAn copolymer

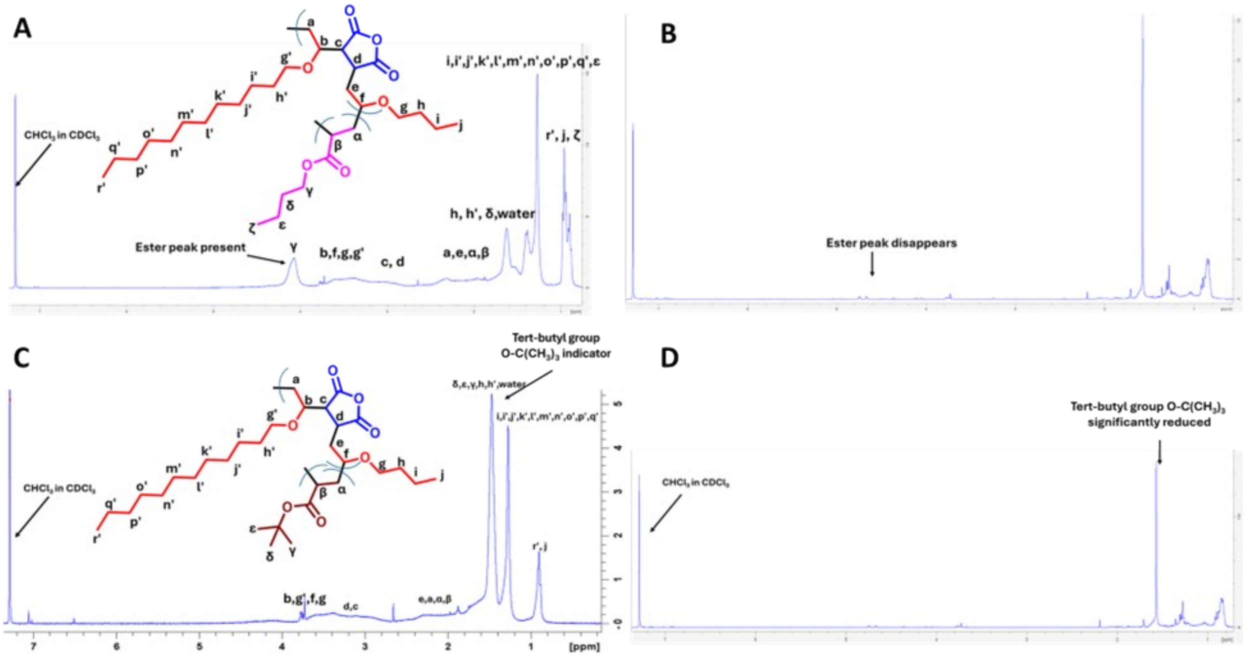

Figure S3. Proton -NMR of A) VEMA-block-nBA (Before Hydrolysis) B) VEMA-block-nBA (After Hydrolysis) C) VEMA-block-tBA (Before Hydrolysis) D) VEMA-block-tBA (After Hydrolysis)

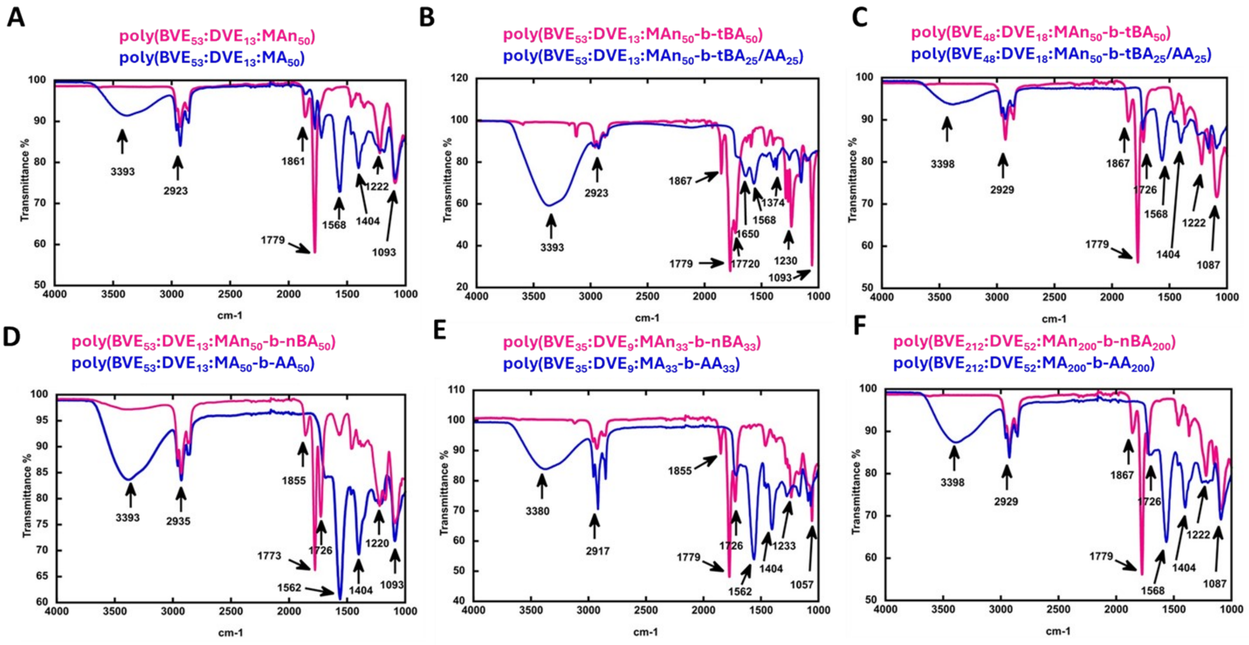

Figure S4. FTIR traces of (before and after hydrolyzing) VEMA copolymers. (A) poly(BVE_{53}:DVE_{13}:MA_{50}) (B) poly(BVE_{53}:DVE_{13}:MA_{50}-b-tBA_{25}/AA_{25}) (C) poly(BVE_{48}:DVE_{18}:MA_{50}-b-tBA_{25}/AA_{25}) (D) poly(BVE_{53}:DVE_{13}:MA_{50}-b-AA_{50}) (E) poly(BVE_{35}:DVE_{9}:MA_{33}-b-AA_{33}) (F) poly(BVE_{212}:DVE_{52}:MA_{200}-b-AA_{200})

Table S1. Functional group analysis of FTIR traces:

Wavenumber (cm^{-1})	Pink Spectrum (Before Hydrolysis)	Blue Spectrum (After Hydrolysis)	Functional Group Change
3350-3400^	Absent/ weak, broad peak	Strong, broad peak	**O-H stretching**: Confirm the formation of carboxylic acid (Maleic acid) groups after hydrolysis.[3]
2917-2929	Strong peak	Strong peak	**sp^3 C-H stretching:** Unchanged sp^3 C-H stretch.[3]
1855-1867, 1773-1779	Two strong sharp peaks	Peaks disappear	**Asymmetric and symmetric C=O stretch:** Disappearance of Maleic anhydride peaks confirm complete hydrolysis.[3]
~1730	Sharp peak present in all spectra	Peak in all spectra	**Esters & Carboxylic Acid C=O**: The presence of a peak in unhydrolyzed samples confirms the existence of esters. All hydrolyzed spectra show this peak consistent with carboxylic acid **C=O**.[3]
1550-1650	Absent	Present	**Carboxylate COO^-**: COO^- (carboxylic acid salt) present in hydrolyzed polymers.[3]
1374-1568	Absent/weak peak	Two strong sharp peaks	**Asymmetric and symmetric COO^- stretch:** Appearance of COO^- confirms hydrolysis.[3]
1222-1233, 1057-1093	Present	Present	**Asymmetric and symmetric C-O-C stretching:** Confirms C-O-C presence of ethers (BVE and DVE) in polymers, and also the esters.[3,4]

^ Trace amounts of water cannot be fully excluded.

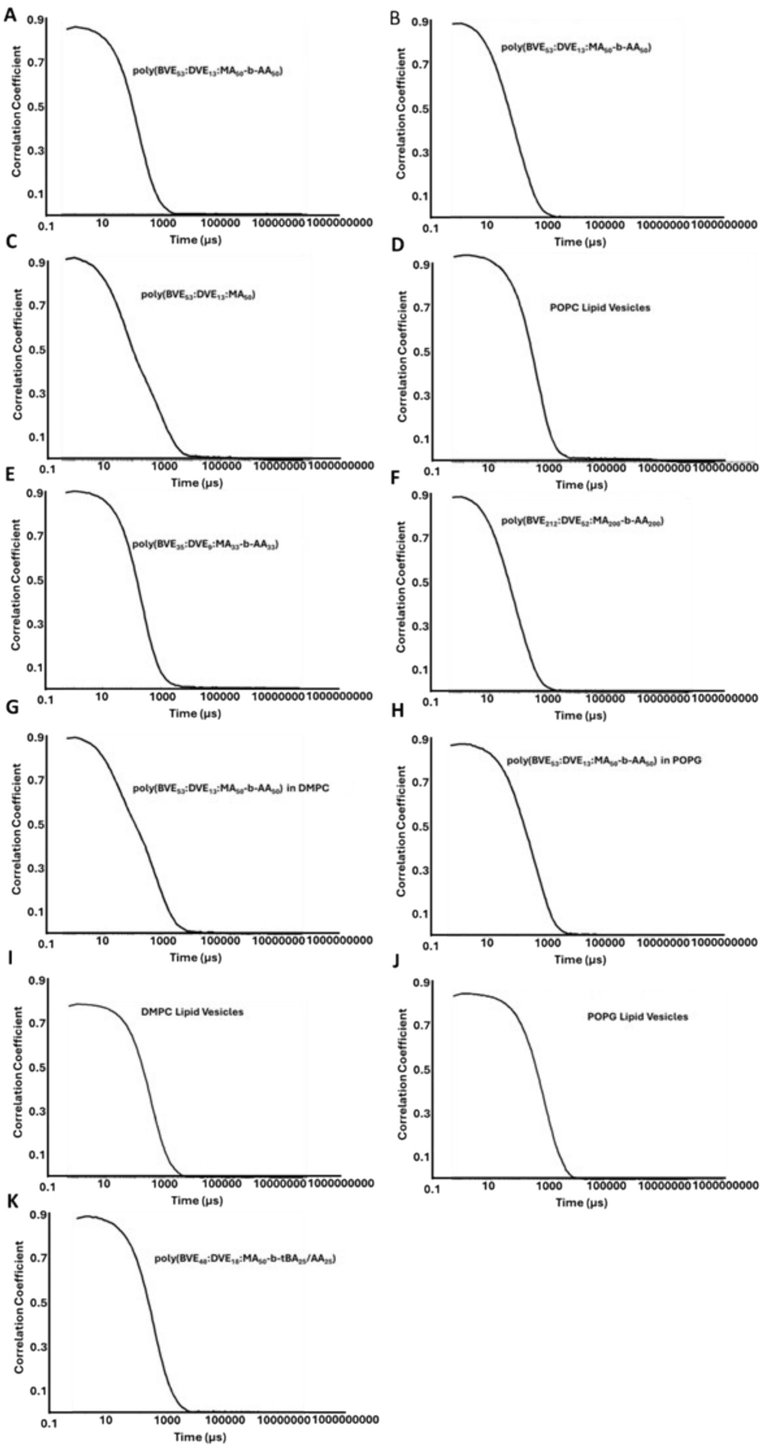

Figure S5. (A-K) Autocorrelation plots for the DLS results for Figure 4.

DOSY NMR:

A

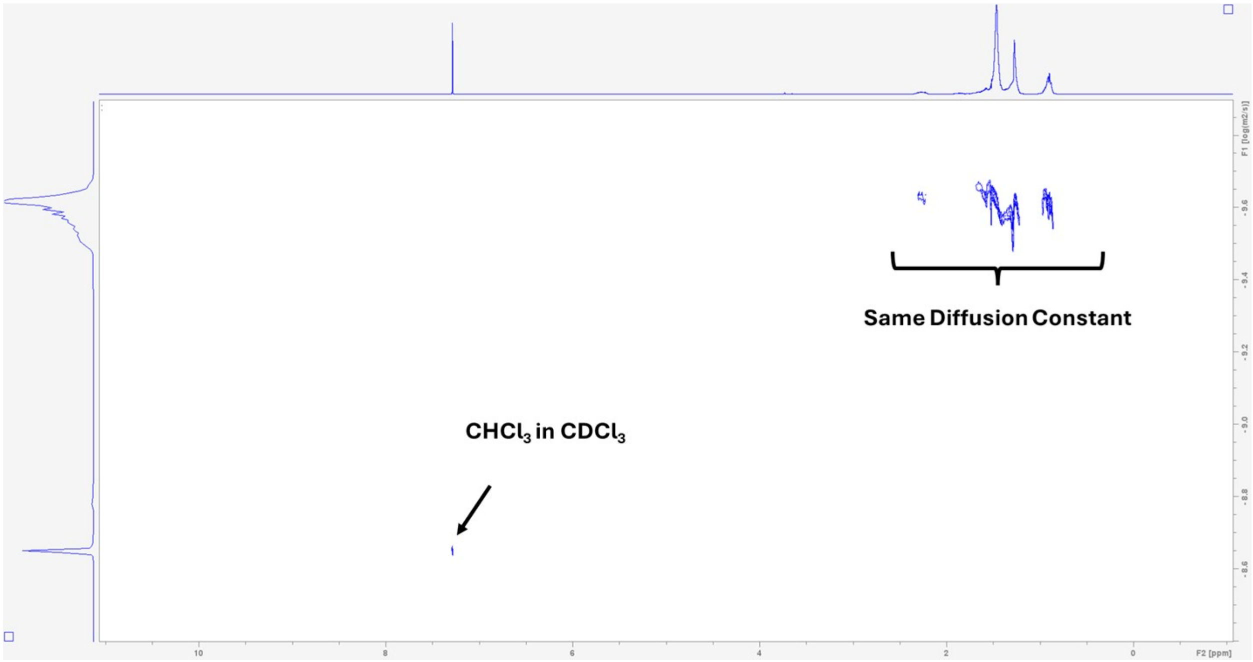

B

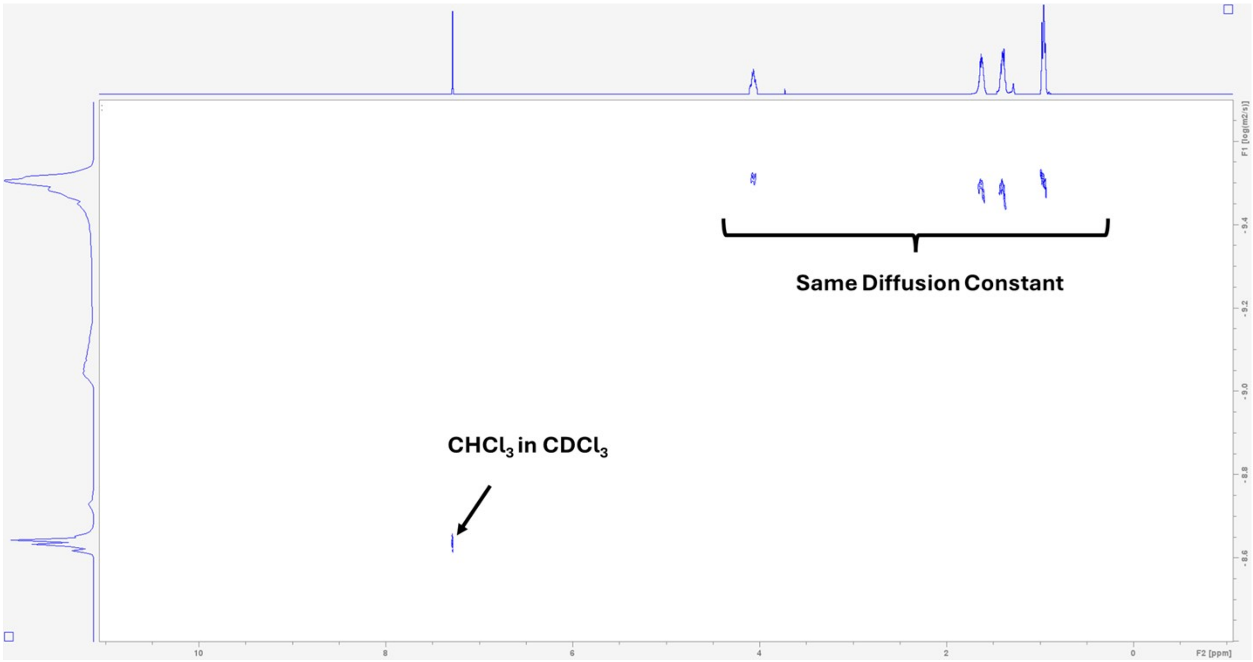

Figure S6. DOSY NMR of A) VEMA-block-tBA Copolymer B) VEMA-block-nBA Copolymer

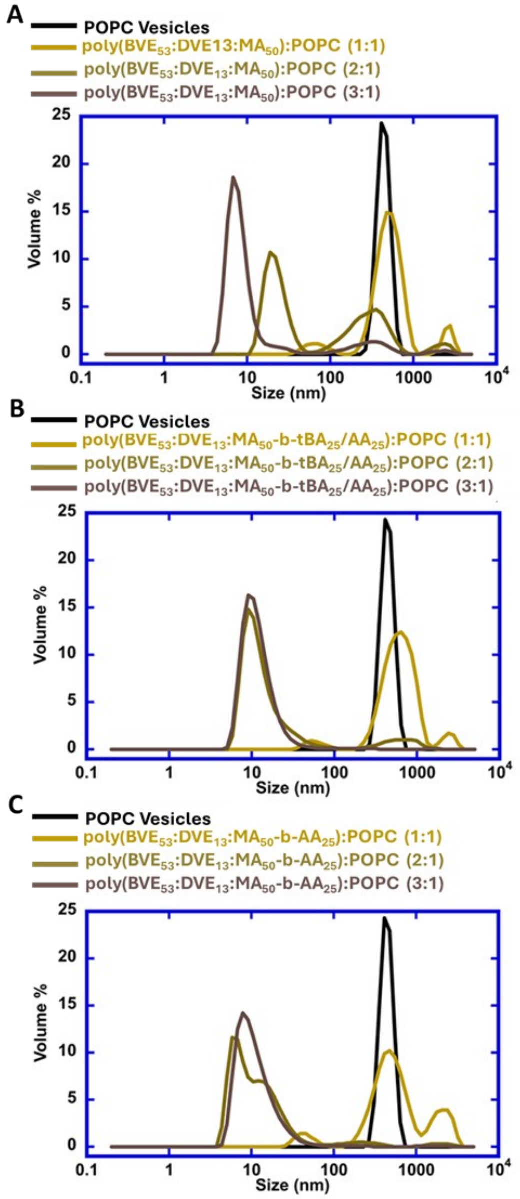

Figure S7. DLS results of polymer based lipid discs by using variable lipid-polymer ratios 1:1, 1:2, and 1:3 for A) poly(BVE_{53}:DVE_{13}:MAn_{50}) Copolymer B) poly(BVE_{53}:DVE_{13}:MA_{50}-b-tBA_{25}/AA_{25}) Copolymer C) poly(BVE_{53}:DVE_{13}:MA_{50}-b-AA_{50}) Copolymer

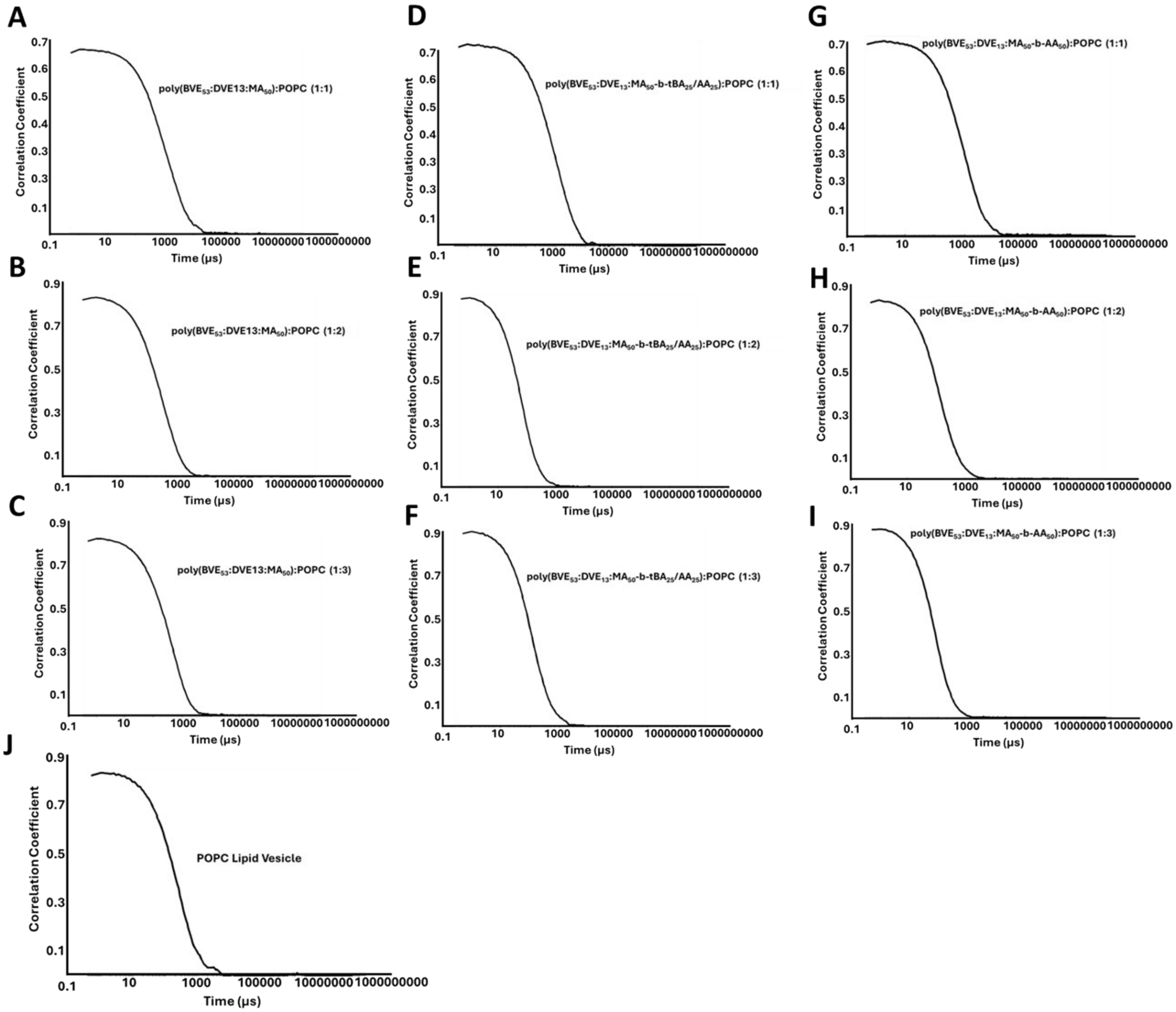

Figure S8. A-J) DLS results of correlation coefficient plots of polymer based lipid discs used in Figure S7 based on poly(BVE_{53}:DVE_{13}:MAn_{50}), poly(BVE_{53}:DVE_{13}:MA_{50}-b-tBA_{25}/AA_{25}) Copolymer and poly(BVE_{53}:DVE_{13}:MA_{50}-b-AA_{50}) Copolymers

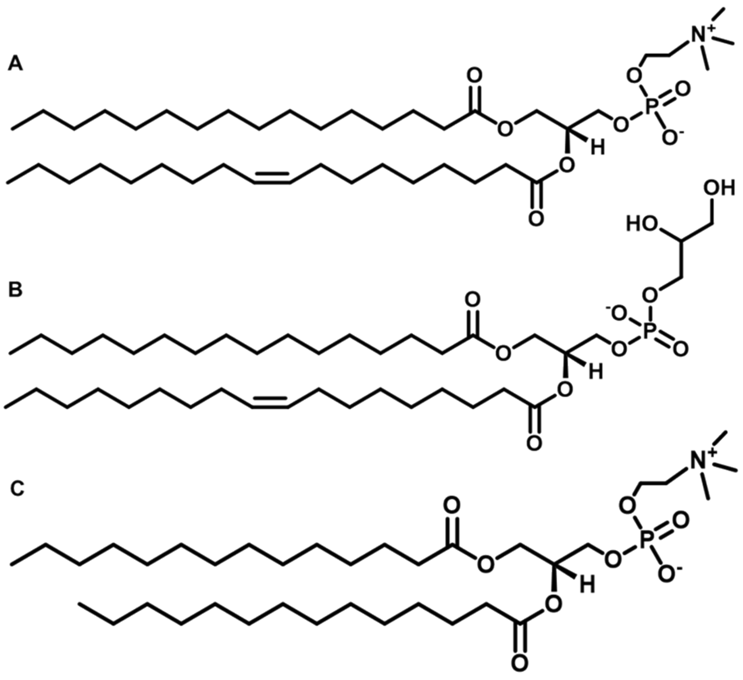

Figure S9. Lipid structures of A) 1-palmitoyl-2-oleoyl-sn-glycero-3-phosphocholine (POPC) B) 1-palmitoyl-2-oleoyl-sn-glycero-3-phospho-(1'-rac-glycerol) (POPG) C) 1,2-Dimyristoyl-sn-glycero-3-phosphocholine (DMPC)

A

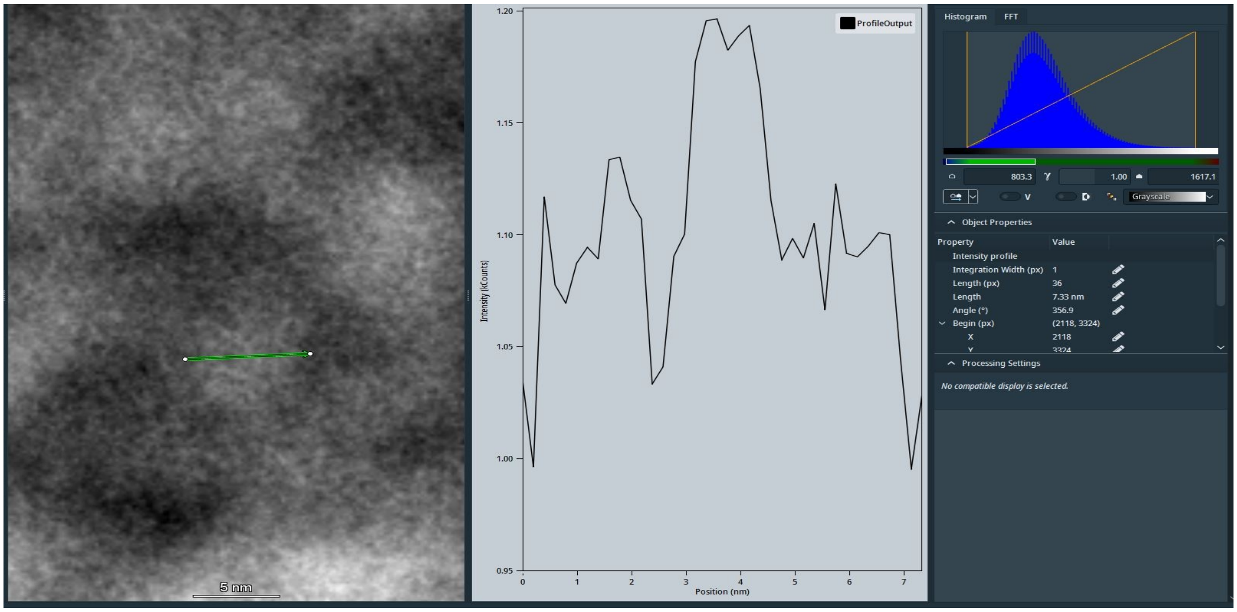

5 nm
ProfileOutput
Intensity (kCounts)
Position (nm)
Histogram
FFT
803.3
1.00
1617.1
Grayscale
Object Properties
Property
Value
Intensity profile
Integration Width (px) 1
Length (px) 36
Length 7.33 nm
Angle (°) 356.9
Begin (px) (2118, 3324)
X 2118
Processing Settings
No compatible display is selected.

B

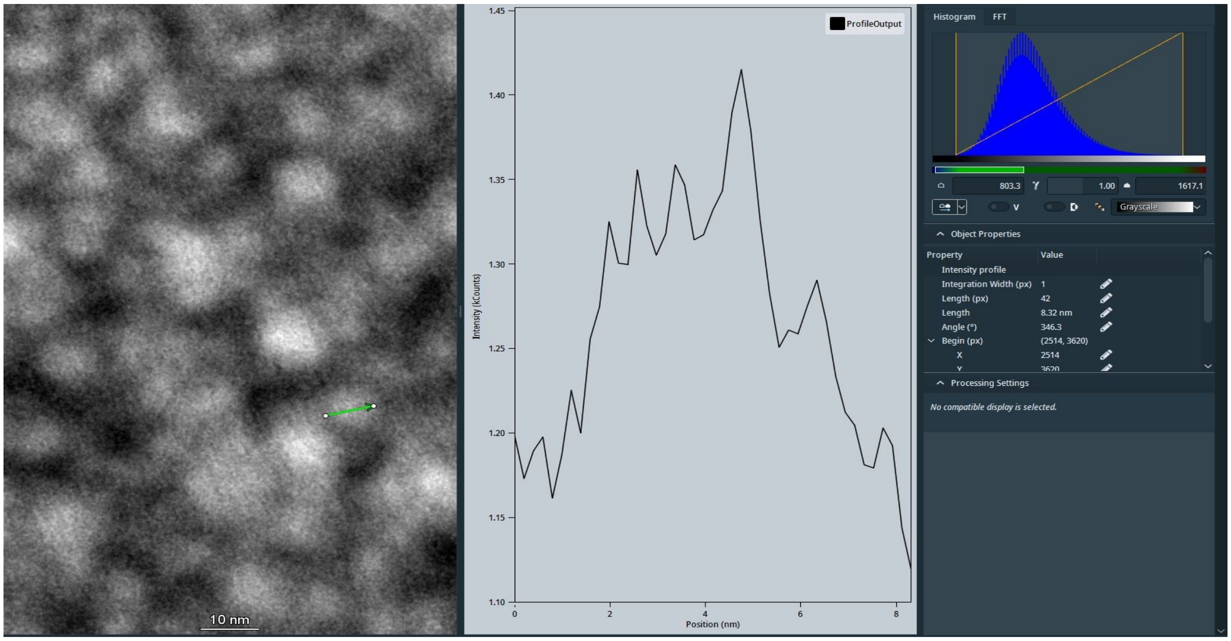

10 nm
ProfileOutput
Intensity (kCounts)
Position (nm)
Histogram
FFT
803.3
1.00
1617.1
Grayscale
Object Properties
Property
Value
Intensity profile
Integration Width (px) 1
Length (px) 42
Length 8.32 nm
Angle (°) 346.3
Begin (px) (2514, 3620)
X 2514
Processing Settings
No compatible display is selected.

C

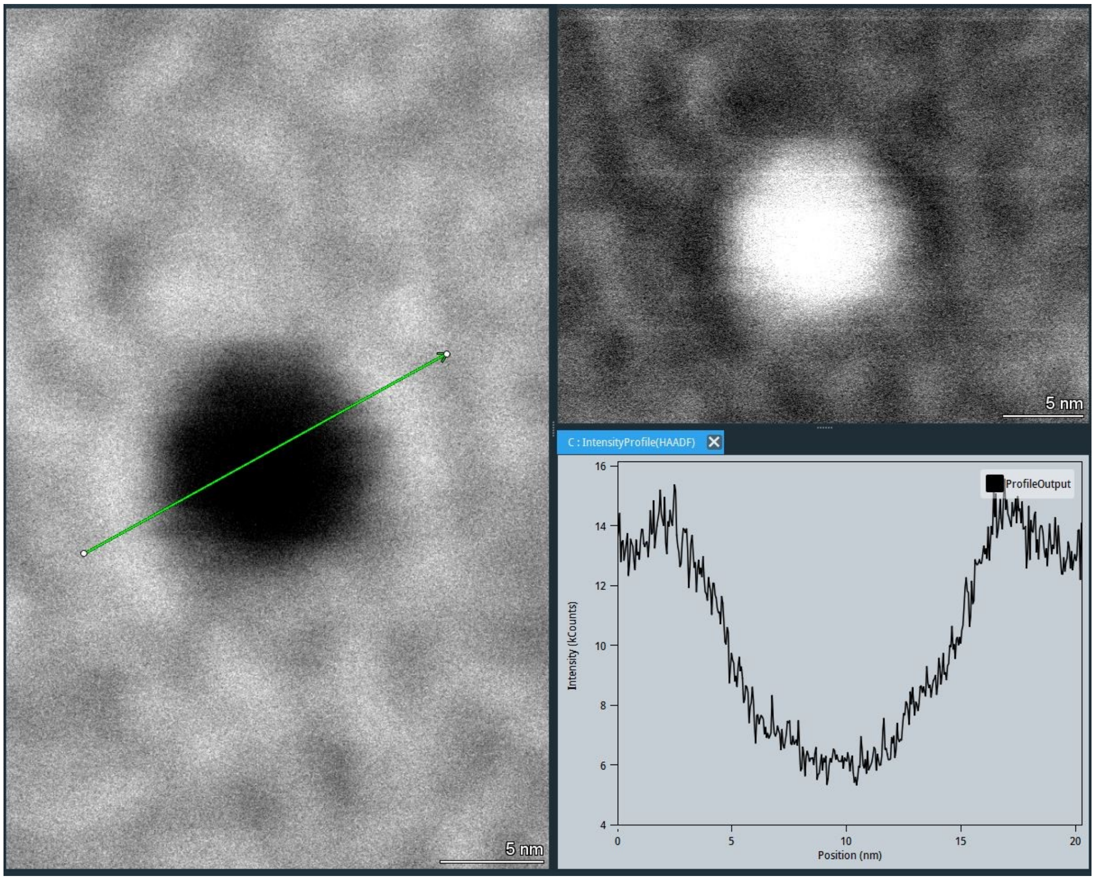

Figure S10. poly(BVE_{53}:DVE_{13}:MA_{50}-b-AA_{50}) based lipid discs TEM images. A-C) Intensity profile of lipid discs.

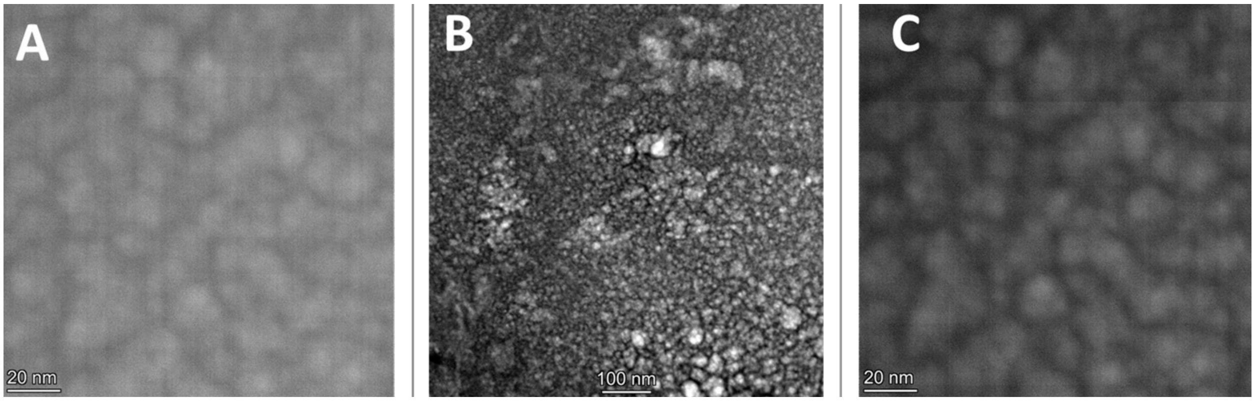

Figure S11. poly(BVE_{53}:DVE_{13}:MA_{50}-b-AA_{50}) based lipid discs TEM images at A) 20nm scale B) 100nm scale C) 20nm scale

References:

(1) Shah, M. Z.; Rotich, N. C.; Okorafor, E. A.; Oestreicher, Z.; Demidovich, G.; Eapen, J.; Henoch, Q.; Kilbey, J.; Prempeh, G.; Bates, A.; Page, R. C.; Lorigan, G. A.; Konkolewicz, D. Vinyl Ether Maleic Acid Polymers: Tunable Polymers for Self-Assembled Lipid Nanodiscs and Environments for Membrane Proteins. *Biomacromolecules* **2024**. https://doi.org/10.1021/acs.biomac.4c00772.

(2) Bradford, K. G. E.; Petit, L. M.; Whitfield, R.; Anastasaki, A.; Barner-Kowollik, C.; Konkolewicz, D. Ubiquitous Nature of Rate Retardation in Reversible Addition–Fragmentation Chain Transfer Polymerization. *J. Am. Chem. Soc.* **2021**, *143* (42), 17769–17777. https://doi.org/10.1021/jacs.1c08654.

(3) Coates, J. Interpretation of Infrared Spectra, a Practical Approach. *Encycl. Anal. Chem.* **2000**, *12*, 10815–10837.

(4) Smith, B. C. The Infrared Spectra of Polymers, VI: Polymers With C-O Bonds. *Spectroscopy* **2022**, 15-19,27. https://doi.org/10.56530/spectroscopy.ly3071f5.
